# Simulating the non-Hermitian dynamics of financial option pricing with quantum computers

**DOI:** 10.1038/s41598-025-97245-3

**Published:** 2025-04-17

**Authors:** Swagat Kumar, Colin Michael Wilmott

**Affiliations:** 1 Department of Mathematics, Nottingham Trent University, Nottingham, NG11 8NS UK; 2 INSAIT, Sofia University “St. Kliment Ohridski”, Sofia, Bulgaria

**Keywords:** Quantum simulation, Quantum information, Qubits

## Abstract

The Schrödinger equation describes how quantum states evolve according to the Hamiltonian of the system. For physical systems, we have it that the Hamiltonian must be a Hermitian operator to ensure unitary dynamics. For anti-Hermitian Hamiltonians, the Schrödinger equation instead models the evolution of quantum states in imaginary time. This process of imaginary time evolution has been used successfully to calculate the ground state of a quantum system. Although imaginary time evolution is non-unitary, the normalised dynamics of this evolution can be simulated on a quantum computer using the quantum imaginary time evolution (QITE) algorithm. In this paper, we broaden the scope of QITE by removing its restriction to anti-Hermitian Hamiltonians, which allows us to solve any partial differential equation (PDE) that is equivalent to the Schrödinger equation with an arbitrary, non-Hermitian Hamiltonian. An example of such a PDE is the famous Black-Scholes equation that models the price of financial derivatives. We will demonstrate how our generalised QITE methodology offers a feasible approach for real-world applications by using it to price various European option contracts modelled according to the Black-Scholes equation.

A financial derivative is an options contract whose value derives from an underlying financial asset^1^. An options contract defines an agreement between two parties that entails a right to trade an asset at some specified future date for a fixed price. This right to trade agreement thus creates inherent value, which may in turn be traded in the same manner as the underlying financial asset. Consequently, a financial derivative may be viewed as an instrument, which can be used to either exploit arbitrage opportunities or mitigate risk exposure in the market. For this reason, a fundamental task in quantitative finance is how exactly do we determine the fair price of a financial derivative. Determining the fair price of an option is a highly non-trivial task, which is due in part to the stochastic nature of the parameters that define a derivative. While classical approaches are computationally-intensive and possess inherent limitations, quantum computing is an emerging technology that has the potential to transform industry, including financial technology. Quantum computing could prove crucial for simulating computationally-complex financial derivatives, and help reduce complexities associated with trading environments.

The famous Black-Scholes model^1,2^ is an effective method for determining the fair price of a derivative, and has become the standard for pricing European style financial options. Given the payoff price for an option at the maturity time, we can determine the present price of the option by solving the linear differential equation

1 $$\begin{aligned} \frac{\partial u}{\partial t} = -\frac{1}{2}(\sigma x)^2 \frac{\partial ^{2}u}{\partial x^{2}} -rx \frac{\partial u}{\partial x} +ru, \end{aligned}$$

for $(x,t) \in [x_0,x_N] \times [0,T]$, where the condition $u(x,T) = p(x)$ denotes the payoff of the option. The price of the option is denoted by *u*(*x*, *t*), while *x* denotes the value of the underlying asset, *t* represents time, and *T* is the maturity time. For simplicity, it is assumed that the volatility of the asset, $\sigma$, and the risk-free interest rate, *r*, are constant with respect to time. For convenience, adopting $\tau = T-t$ transforms the Black-Scholes equation Eq. (1) to the initial value problem

2 $$\begin{aligned} \frac{\partial u}{\partial \tau } = \frac{1}{2}(\sigma x)^2 \frac{\partial ^{2}u}{\partial x^{2}} +rx \frac{\partial u}{\partial x} -ru, \end{aligned}$$

for $(x,\tau ) \in [x_0,x_N] \times [0,T]$ with the initial condition $u(x,\tau =0) = p(x)$. To numerically solve the Black-Scholes equation, we must discretise the domain $[x_0,x_N]$ to a finite domain and assign appropriate boundary conditions.

The Schrödinger equation models the evolution of the wave function of a quantum mechanical system, and takes the form

3 $$\begin{aligned} i \frac{\partial \psi (\vec {x},t)}{\partial t} = \hat{H}\psi (\vec {x},t), \end{aligned}$$

where the Hamiltonian, $\hat{H}$, is a linear differential operator in $\vec {x}$ acting on the wave function $\psi$. Solutions to the Schrödinger equation are expressed in terms of the time evolution operator,

4 $$\begin{aligned} \psi (\vec {x},t) = e^{-i\hat{H}t} \psi (\vec {x},0). \end{aligned}$$

The Black-Scholes equation, Eq. (2), can also be expressed in the form of the Schrödinger equation, where its Hamiltonian is given by

5 $$\begin{aligned} \hat{H}_{BS} = i\left[ \frac{1}{2}(\sigma x)^2 \frac{\partial ^{2}}{\partial x^{2}} + rx \frac{\partial }{\partial x} - r\right] . \end{aligned}$$

Note that while the Hamiltonian of the Schrödinger equation is a Hermitian operator, which gives rise to unitary time evolution, the Black-Scholes Hamiltonian, Eq. (5), is non-Hermitian, and induces non-unitary time evolution. However, since quantum computers evolve under unitary time evolution, it is the case that simulating non-Hermitian dynamics is not directly feasible on a quantum computer. It is for this reason that quantum computing approaches for solving the Black-Scholes equation have thus far relied on approximating non-unitary time evolution with unitary operators^3–6^.

Non-Hermitian Hamiltonians found use in modelling open quantum systems^7^ and pseudo-Hermitian, in particular PT-symmetric, Hamiltonians have been explored as candidates to generalise the theory of quantum mechanics^8,9^. Another example of non-Hermitian dynamics can be seen in the imaginary time evolution of a quantum system. Following a Wick rotation, which replaces time with an imaginary number $\beta =it$, the Schrödinger equation drives wave functions to become parallel to the ground state of the system. The Wick-rotated form of the Schrödinger equation also takes the form of Eq. (3), but with an anti-Hermitian Hamiltonian. Although the imaginary time Schrödinger equation induces non-unitary dynamics, the normalised evolution can be simulated with quantum algorithms, including quantum imaginary time evolution (QITE)^10^ and variational QITE^11^.

Variational QITE (varQITE) is a hybrid quantum-classical algorithm that is well suited for noisy intermediate-scale quantum (NISQ) devices. As a variational quantum algorithm, varQITE considers a system of differential equations linking to the gradients of ansatz parameters in imaginary time, and coefficients that depend on measurements of the ansatz. Variational QITE employs a fixed ansatz, where the time complexity is linear in the number of Hamiltonian terms. However, the choice of ansatz is crucial, as it is possible that the states produced by the true imaginary time evolution may not be generated by the particular parameterised ansatz circuit. VarQITE has been used to indirectly simulate Black-Scholes through a change of variables, $x=e^s$, which transforms $\hat{H}_{BS}$ into an anti-Hermitian Hamiltonian^3–5^. Solutions to the original Black-Scholes equation are obtained by undoing the variable change on the solutions obtained from QITE.

On the other hand, the simulated QITE approach is an alternative technique for simulating imaginary time evolution. The technique makes use of the Trotter product approximation, which approximates the normalised imaginary time evolution with a product of unitary operators acting on local neighbourhoods of qubits. Simulated QITE with sufficiently large unitary domains is not plagued by barren plateaus, as is the case with its variational counterpart. Simulated QITE on a *k*-local Hamiltonian requires a number of measurements that is exponential in *k*, with the depth of the associated quantum circuits scaling accordingly. Interestingly, however, recent work has focused on optimising the circuit depth and the number of measurements required in simulated QITE. For instance, Fast QITE provides for an exponential reduction in the circuit depth of each unitary and also reduces the number of measurements required per time step, leading to a quadratic speedup over QITE^12^. A time dependent drifted QITE introduces the concept of randomised compiling, which reduces the unitary circuit depth to be a constant and also reduces the number of measurements needed^13^. We also have an implementation of QITE using nonlocal approximation, which reduces circuit depth and is NISQ-friendly^14^.

Although imaginary time evolution was originally envisioned as a technique for determining the ground state of a Hamiltonian^10,11^, the methodology has been recently used as an approach for solving partial differential equations (PDEs), primarily based on varQITE ^3,5,15–17^. A simulated QITE approach for solving linear PDEs has also been considered^18^, however the approach is restricted to anti-Hermitian Hamiltonians involving only even-ordered derivatives. In particular, the technique tracks how the non-unitary time evolution scales the quantum state over time, and the approach was used to generate solutions to the isotropic heat equation by combining the scale information with the normalised states obtained from QITE.

In this paper, we further widen the scope of simulated QITE by broadening the methodology to simulations involving arbitrary non-Hermitian dynamics. By removing simulated QITE’s restriction to anti-Hermitian Hamiltonians, we enhance the capabilities of the methodology with an ability to simulate arbitrary linear PDEs involving non-unitary time evolution. We have called this generalisation of simulated QITE to arbitrary Hamiltonians quantum non-unitary time evolution (QNUTE).

## Results

### Quantum non-unitary time evolution

QNUTE is a quantum algorithm that simulates the dynamics of the Schrödinger equation with an arbitrary non-Hermitian Hamiltonian ${\hat{H} = \sum _{m=1}^M i\hat{h}_m}$, where each $\hat{h}_m$ is a local operator which may be non-Hermitian. The non-unitary time evolution operator generated by $\hat{H}$ is approximated by its first order Trotter product, and takes the form

6 $$\begin{aligned} e^{-i\hat{H}T} \approx \left( \prod _{m=1}^M e^{\hat{h}_m \Delta t}\right) ^{N_T}, \end{aligned}$$

where $N_T = T/\Delta t$^19,20^. The normalised actions of each Trotter step $e^{\hat{h}_m\Delta t}$ acting on a state $\vert \psi \rangle$ are approximated with unitaries of the form $e^{-i\hat{A}\Delta t}$, and implemented with Trotter products of the form

7 $$\begin{aligned} e^{-i\hat{A}\Delta t} \approx \prod _{I=1}^{\mathcal {I}} e^{-i a_I \hat{\sigma }_I \Delta t}. \end{aligned}$$

In Eq. (7), ${\hat{A}=\sum _{I=1}^{\mathcal {I}} a_I \hat{\sigma }_I}$ is a Hermitian operator with $\hat{\sigma }_I$ denoting Hermitian operators chosen such that each unitary $e^{-i \theta \hat{\sigma }_I }$ is efficiently implemented with a quantum circuit parameterised by $\theta$. The real-valued coefficients $a_I$ are determined by minimising the expression

8 $$\begin{aligned} \left\| \frac{e^{\hat{h}_m \Delta t}\vert \psi \rangle }{\sqrt{ \langle {\psi }\vert {e^{\hat{h}_m^\dagger \Delta t} e^{\hat{h}_m \Delta t}}\vert {\psi }\rangle }} - e^{-i\hat{A}\Delta t}\vert \psi \rangle \right\| , \end{aligned}$$

up to $O(\Delta t)$, which involves solving a system of linear equations, ${(S+S^\top )\, \vec {a}=\vec {b}}$, constructed using various measurements on $\vert \psi \rangle$. In particular, we have

9 $$\begin{aligned} S_{I,J} = \langle {\psi }\vert {\hat{\sigma }_I^\dagger \hat{\sigma }_J}\vert {\psi }\rangle , \quad c = \sqrt{1 + 2\Delta t\, \text {Re} \langle {\psi }\vert {\hat{h}_m}\vert {\psi }\rangle }, \quad b_I = \frac{-2}{c}\, \text {Im} \langle {\psi }\vert {\hat{\sigma }_I^\dagger \, \hat{h}_m}\vert {\psi }\rangle , \end{aligned}$$

see Supplementary Information for further details on the construction. Simulating each Trotter step involves taking $O(\mathcal {I}^2)$ measurements to construct the $\mathcal {I}\times \mathcal {I}$ matrix equation and generates a quantum circuit of depth $O(\mathcal {I})$. The full simulation therefore requires $O(N_T M \mathcal {I}^2)$ measurements.

The states generated by QNUTE are determined by the choice of $\hat{\sigma }_I$. For example, choosing $\hat{\sigma }_I$ to encompass all Pauli strings allows us to capture arbitrary state vector rotations in the state space, whereas restricting $\hat{\sigma }_I$ to Pauli strings involving an odd number of $\hat{Y}$ gates significantly reduces the operator decomposition count and allows us to capture those rotations that do not introduce complex phases to the quantum state. Given a choice of $\hat{\sigma }_I$, the accuracy of the QNUTE implementation is dependent on the support of $\hat{A}$. Ideally, the support of $\hat{A}$ should cover ${D=O(C)}$ adjacent qubits surrounding the support of $\hat{h}_m$, where the correlation length *C* denotes the maximum distance between interacting qubits in the Hamiltonian. However, our choice to express $\hat{A}$ has been in terms of Pauli strings, which gives rise to an exponential dependence on *D*, ${\mathcal {I}=O(2^D)}$. For this reason, we have considered an inexact implementation of QNUTE that uses a constant domain size $D<C$.

We will demonstrate that QNUTE can be used to approximate solutions to arbitrary linear PDEs with solutions stored in the qubit state vector. Information relevant to the solution is extracted by taking measurements on the final quantum state. It is expected that the number of distinct measurements required to extract the relevant information should scale polynomially with the number of qubits. Further, if it is known that the solution to a PDE will be real-valued and non-negative, then the normalised solution calculated by QNUTE can be extracted obtained by taking the square root of the probability distribution of computational basis states. We will use QNUTE to simulate the Black-Scholes equation, as it has a non-Hermitian Hamiltonian and has non-negative real-valued solutions.

### Simulating black-scholes with QNUTE

To model the dynamics of the Black-Scholes equation, we discretise the domain $[x_0,x_N]$ into $2^n$ points equally spaced by a distance of $h = \frac{x_N - x_0}{2^n - 1}$. The normalised samples of the option price are stored in an *n*-qubit quantum state given by

10 $$\begin{aligned} \vert \bar{u}(\tau )\rangle = \frac{\sum _{k=0}^{2^n-1} u(x_k, \tau ) \vert k\rangle }{\sqrt{\sum _{k=0}^{2^n-1} u^2(x_k, \tau ) }}, \end{aligned}$$

where $x_k = x_0 + kh$. Following Eq. (5), the discretised Black-Scholes Hamiltonian can be represented in terms of a central finite difference matrix of the form

11 $$\begin{aligned} -i\hat{H}_{BS} = \begin{bmatrix} \gamma _0 & \beta _0 & & \\ \alpha _1 & \gamma _1 & \beta _1 & \\ & \ddots & \ddots & \ddots & & \\ & & \alpha _{2^n-2} & \gamma _{2^n-2} & \beta _{2^n-2} \\ & & & \alpha _{2^n-1} & \gamma _{2^n-1} \end{bmatrix}, \end{aligned}$$

where

12 $$\begin{aligned} \alpha _k = \frac{\sigma ^2 x_k^2}{2h^2} - \frac{r x_k}{2h}, \quad \beta _k = \frac{\sigma ^2 x_k^2}{2h^2} + \frac{r x_k}{2h} \quad \text {and} \quad \gamma _k = -r - \alpha _k - \beta _k. \end{aligned}$$

Refer to Supplementary Information for the representation of the discretised Hamiltonian of Eq. (11) in the Pauli operator basis.

### Norm correction

The scale factor *c* given in Eq. (9) approximates how the Trotter step scales $\vert \psi \rangle$ up to $O(\Delta t)$. These approximations can be stored and multiplied to provide an approximation of how the state vector scales over the course of the evolution. Excluding the scenario of the ideal implementation of QNUTE that records a perfect fidelity, errors associated to each scale factor will compound over multiple Trotter steps, which must be corrected periodically. For an anti-Hermitian Hamiltonian $\hat{H}=i\hat{L}$, it was shown that the correction factor can be calculated using knowledge of the non-degenerate ground state $\vert \psi _0\rangle$ of $\hat{L}$ and its corresponding eigenvalue $\lambda _0$^18^. This correction strategy necessarily exploits the mutual orthogonality of the eigenstates of the associated Hamiltonian.

However, since the discretised Black-Scholes Hamiltonian as given in Eq. (11) is not a normal operator, its eigenvectors are not guaranteed to be mutually orthogonal. This, therefore, rules out the norm correction strategy pursued in Ref.^18^. Interestingly, variational QITE has been employed as a technique for solving the Black-Scholes equation. Under this setting, the normalisation factor was considered either as a variational parameter^5^ or was determined with prior knowledge of how, specifically, call option prices evolve at the boundary $x_N$^3^. Since the former is not compatible with QNUTE, we generalise the latter approach to cater to various European option types.

Consider the Black-Scholes equation, as given in Eq. (5), with option price $u(x,\tau )$ assumed to be linear in *x* in the neighbourhood of the boundaries $x_0$ and $x_N$. We will consider linear boundary conditions, since they are widely used in classical option pricing simulations and are known to be numerically stable^21^. Thus, under linear boundary conditions, the option price takes the form ${u(x,\tau ) = a(\tau )x + b(\tau )}$ near the boundaries. Substituting this form into Eq. (5) reduces the Black-Scholes equation to an ordinary differential equation (ODE) at the boundaries

13 $$\begin{aligned} x\frac{{\textrm{d}}a}{{\textrm{d}}\tau } + \frac{{\textrm{d}}b}{{\textrm{d}}\tau } = -rb(\tau ). \end{aligned}$$

Solving Eq. (13) yields $a(\tau ) = a(0)$ and $b(\tau ) = b(0)e^{-r\tau }$, where *a*(0), and *b*(0) can be derived from the initial conditions *p*(*x*) at each boundary. If *a*(0) or *b*(0) are non-zero on at least one of the boundaries, we can rescale a normalised solution to ensure that the value at that boundary is equal to $a(\tau )x+b(\tau )$.

To guarantee that the linear boundary conditions apply during the QNUTE simulation, they must be encoded into the Black-Scholes Hamiltonian. The first and last rows of the matrix in Eq. (11) are updated with the corresponding forward and backward first-order finite difference coefficients, respectively, with the second-derivative terms vanishing as the function is linear. The Black-Scholes Hamiltonian inclusive of linear boundary conditions takes the form

14 $$\begin{aligned} -i\hat{H}_{LBS} = \begin{bmatrix} \gamma _0^\prime & \beta _0^\prime & & \\ \alpha _1 & \gamma _1 & \beta _1 & \\ & \ddots & \ddots & \ddots & & \\ & & \alpha _{2^n-2} & \gamma _{2^n-2} & \beta _{2^n-2} \\ & & & \alpha _{2^n-1}^\prime & \gamma _{2^n-1}^\prime \end{bmatrix}, \end{aligned}$$

where

15 $$\begin{aligned} \gamma _0^\prime =\ -r - \frac{r x_0}{h}, \quad \beta _0^\prime =\ \frac{r x_0}{h}, \quad \alpha _{2^n-1}^\prime =\ -\frac{r x_N}{h}, \quad \text {and} \quad \gamma _{2^n-1}^\prime =\ -r + \frac{r x_N}{h}. \end{aligned}$$

See Supplementary Information for the Pauli decomposition of this Hamiltonian.

**Fig. 1:**
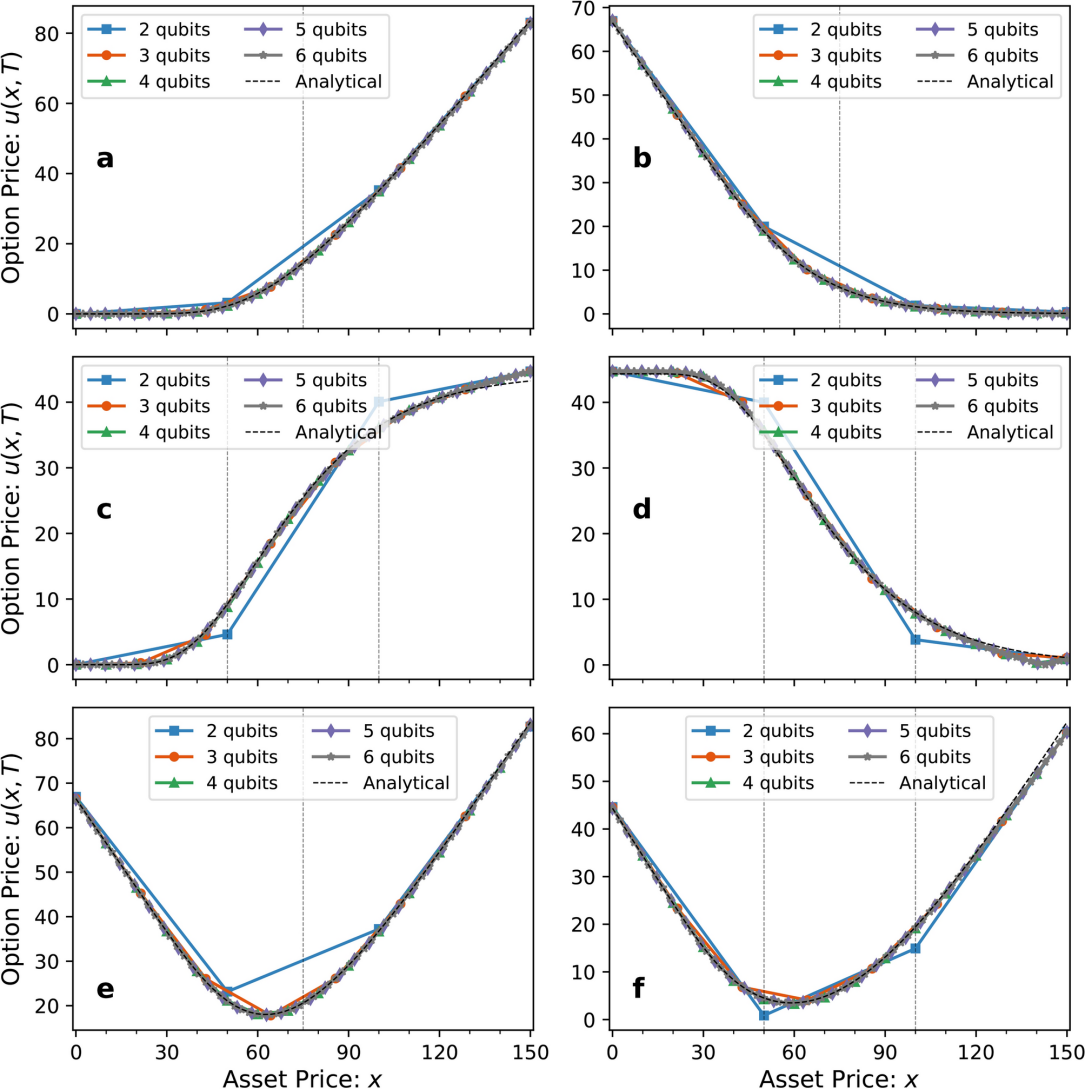
Black-Scholes option pricing simulations using QNUTE. The figure compares the Black-Scholes option prices calculated using QNUTE with varying number of qubits to the corresponding analytical solutions for the following European option types: (**a**) Call (**b**) Put (**c**) Bull Spread (**d**) Bear Spread (**e**) Straddle (**f**) Strangle. The vertical dashed lines at $x=50,75,$ and 100 correspond to the strike prices of the option contracts. We simulated the solutions for the asset prices $x\in [0,150]$, with the maturity time $T=3$ years, simulated over $N_T=500$ time steps. Our simulations used a risk-free interest rate of $r=0.04$, and the volatility $\sigma =0.2$. The unitaries used to approximate the evolution act on all of the qubits used in the simulation.

**Fig. 2:**
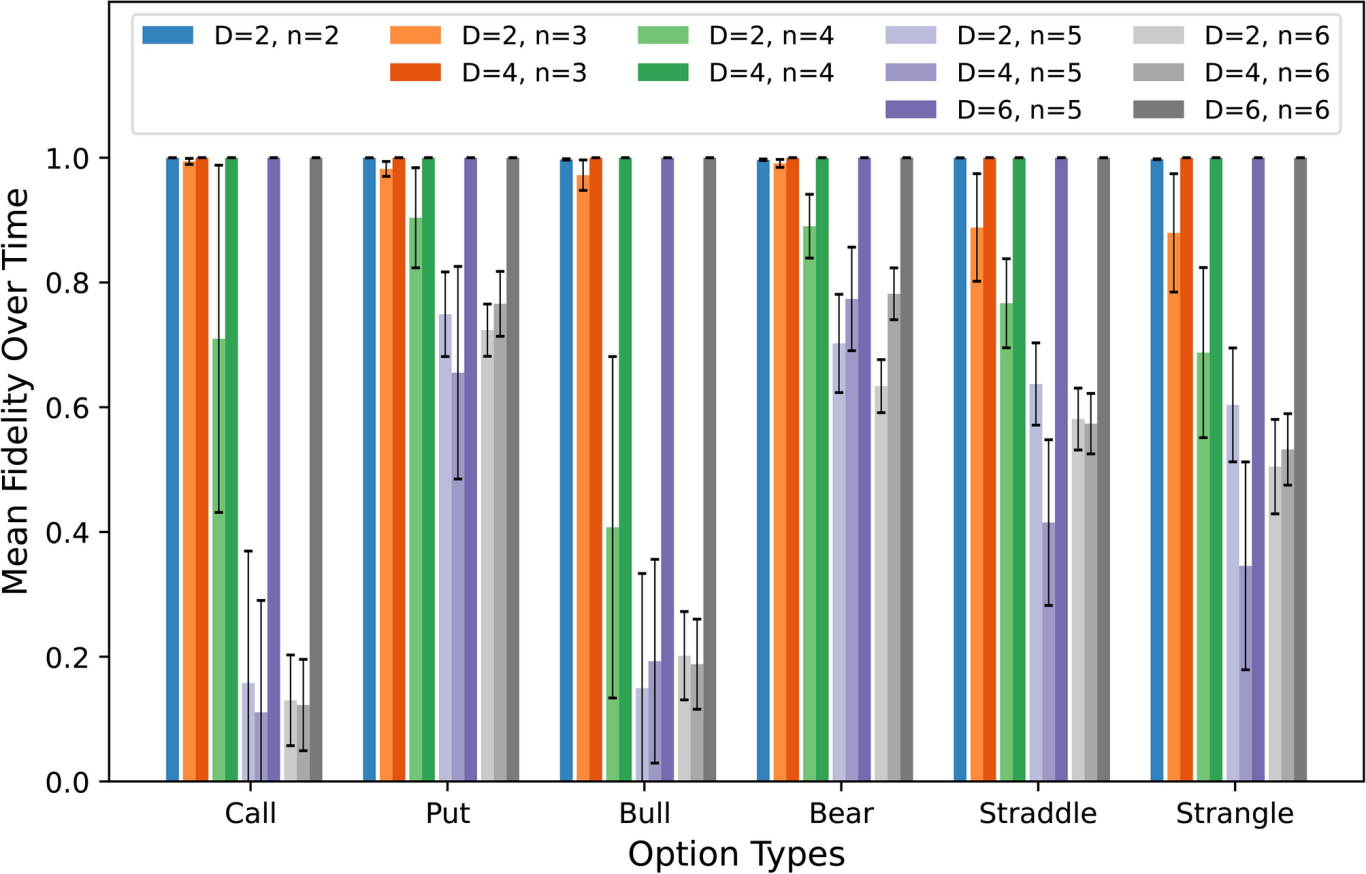
Average fidelities of inexact QNUTE implementations. The figure shows the fidelities of different implementations of inexact QNUTE used to simulate Black-Scholes dynamics averaged over each time step, with the error bars depicting the standard deviation. These simulations share the same parameters values for $r,\sigma ,T$ and $N_T$ as with the simulations shown in Fig. 1. *n* denotes the number of qubits used to store the function samples, and *D* denotes the maximum number of adjacent qubits targeted by the unitaries. The overall low fidelities shown the by inexact QNUTE, where $D<n$, indicate that the Black-Scholes Hamiltonian with linear boundary conditions has a high correlation length, making it difficult to accurately reproduce its evolution with small unitaries.

## Discussion

In this work, we have generalised the quantum imaginary time evolution algorithm to enable the simulation of arbitrary non-Hermitian dynamics on quantum computers. We demonstrated our QNUTE algorithm’s application via a numerical implementation that simulates the pricing dynamics of European options, as dictated by the Black-Scholes equation. Our approach differs from other quantum simulations targetting Black-Scholes as it directly solves the original equation, unlike the methods based on varQITE^3–5^, and it does not rely on post-selection as in Ref.^6^.

In undertaking these simulations, we assumed that the underlying financial asset had constant volatility, $\sigma$, and risk-free interest rate, *r*. The time dependence of these variables can be encoded in the Hamiltonian with no extra cost to its construction. Further, the inclusion of these variables does not affect the unitary approximations produced by QNUTE, however, modelling volatility and interest rates as stochastic processes may require smaller time steps for more accurate simulations. Indeed, the time dependence of *r* gives rise to a different boundary ODE, which necessitates modifications to our rescaling protocol outlined in Supplementary Information.

As depicted in Fig. 1, our implementations of QNUTE were able to match the analytical solutions of the Black-Scholes equation. For convergence, it is important to choose an asset price domain with boundaries such that linear boundary conditions hold for the option’s payoff $u(x,\tau =T)$. A good level of convergence also depends on having access to enough sample points around the strike prices of the option, a lack of which can be seen in the 2-qubit curves in Fig. 1c, d and f.

In our implementations of QNUTE, each term, $\hat{h}_m$, in the decomposition of the Black-Scholes Hamiltonian was a linear combination of Pauli strings. Since the number of distinct Pauli strings in our decomposition scales exponentially with the number of qubits, an alternative decomposition is required for the scalability of QNUTE for Black-Scholes. Approaches taken to solve PDEs using varQITE have also required the expectation values of finite difference operators. In particular, Liu et al. proposed a scheme to measure such expectation values with a linear overhead^17^. We conjecture that this scheme may be adopted within our approach, leading to exponentially fewer terms in the Hamiltonian decomposition.

The discretised Black-Scholes Hamiltonian has a high degree of correlation between all the qubits used in the simulation. This was demonstrated by implementing inexact QNUTE, wherein the unitary approximations only act on at most *D* adjacent qubits. Figure 2 depicts the fidelities of inexact QNUTE simulations, averaged over each time step for the various option payoffs, see Table 1 for the exact values. For an increasing number of qubits and fixed domain size *D*, we have it that inexact QNUTE does not capture the correlations between the qubits, rendering it unable to emulate the true time evolution. For future work, we intend to incorporate recent improvements to the simulated QITE methodology, including Fast QITE^12^, time-dependent drifted QITE ^13^ and QITE with nonlocal approximation^14^, within our QNUTE framework to understand their effect on the accuracy and efficiency for simulated PDE dynamics.

**Table 1:** Average fidelity data for the QNUTE simulations for pricing various European option types.

*n*	*D*	Call		Put	
		$\mu _F$	$\sigma _F$	$\mu _F$	$\sigma _F$
2	2	1.000	2.67$\times 10^{-5}$	1.000	4.54$\times 10^{-5}$
3	2	0.994	4.77$\times 10^{-3}$	0.982	1.20$\times 10^{-2}$
	4	1.000	3.26$\times 10^{-6}$	1.000	3.06$\times 10^{-6}$
4	2	0.710	2.78$\times 10^{-1}$	0.904	8.02$\times 10^{-2}$
	4	1.000	3.16$\times 10^{-7}$	1.000	2.79$\times 10^{-7}$
5	2	0.158	2.11$\times 10^{-1}$	0.749	6.79$\times 10^{-2}$
	4	0.111	1.79$\times 10^{-1}$	0.655	1.70$\times 10^{-1}$
	6	1.000	4.15$\times 10^{-8}$	1.000	2.10$\times 10^{-7}$
6	2	0.130	7.27$\times 10^{-2}$	0.724	4.18$\times 10^{-2}$
	4	0.122	7.32$\times 10^{-2}$	0.766	5.20$\times 10^{-2}$
	6	1.000	3.81$\times 10^{-8}$	1.000	2.18$\times 10^{-7}$

*n*	*D*	Bull		Bear	
		$\mu _F$	$\sigma _F$	$\mu _F$	$\sigma _F$
2	2	1.000	2.67$\times 10^{-5}$	1.000	4.54$\times 10^{-5}$
3	2	0.994	4.77$\times 10^{-3}$	0.982	1.20$\times 10^{-2}$
	4	1.000	3.26$\times 10^{-6}$	1.000	3.06$\times 10^{-6}$
4	2	0.710	2.78$\times 10^{-1}$	0.904	8.02$\times 10^{-2}$
	4	1.000	3.16$\times 10^{-7}$	1.000	2.79$\times 10^{-7}$
5	2	0.158	2.11$\times 10^{-1}$	0.749	6.79$\times 10^{-2}$
	4	0.111	1.79$\times 10^{-1}$	0.655	1.70$\times 10^{-1}$
	6	1.000	4.15$\times 10^{-8}$	1.000	2.10$\times 10^{-7}$
6	2	0.130	7.27$\times 10^{-2}$	0.724	4.18$\times 10^{-2}$
	4	0.122	7.32$\times 10^{-2}$	0.766	5.20$\times 10^{-2}$
	6	1.000	3.81$\times 10^{-8}$	1.000	2.18$\times 10^{-7}$

*n*	*D*	Straddle		Strangle	
		$\mu _F$	$\sigma _F$	$\mu _F$	$\sigma _F$
2	2	1.000	2.67$\times 10^{-5}$	1.000	4.54$\times 10^{-5}$
3	2	0.994	4.77$\times 10^{-3}$	0.982	1.20$\times 10^{-2}$
	4	1.000	3.26$\times 10^{-6}$	1.000	3.06$\times 10^{-6}$
4	2	0.710	2.78$\times 10^{-1}$	0.904	8.02$\times 10^{-2}$
	4	1.000	3.16$\times 10^{-7}$	1.000	2.79$\times 10^{-7}$
5	2	0.158	2.11$\times 10^{-1}$	0.749	6.79$\times 10^{-2}$
	4	0.111	1.79$\times 10^{-1}$	0.655	1.70$\times 10^{-1}$
	6	1.000	4.15$\times 10^{-8}$	1.000	2.10$\times 10^{-7}$
6	2	0.130	7.27$\times 10^{-2}$	0.724	4.18$\times 10^{-2}$
	4	0.122	7.32$\times 10^{-2}$	0.766	5.20$\times 10^{-2}$
	6	1.000	3.81$\times 10^{-8}$	1.000	2.18$\times 10^{-7}$

$\mu _F$ denotes the mean fidelity over each time step and $\sigma _F$ the standard deviation. Simulations involving $D<n$ represent inexact QNUTE implementations.

## Data Availability

All data generated and analysed during this study are included in this published article and its supplementary information files.
